# P-1249. Analysis of Drug Interaction Between Valproic acid and Carbapenems

**DOI:** 10.1093/ofid/ofae631.1431

**Published:** 2025-01-29

**Authors:** Youngmin Cho, Hyejeong Moon, Jaemin Park, Chang Kim, Ji Yeon Han, Hun Min Kim, Yunsang Choi, Seong Jin Choi, Song Mi Moon, Kyoung-Ho Song, Eu Suk Kim, Hong Bin Kim, Woo-Jin Lee, Chang-Ho Yun, Seonghae Yoon, Jae-Yong Chung, Joon Hee Lee, Jeong Su Park, Eunjeong Heo, hyung-Sook Kim, Hyunju Lee

**Affiliations:** Seoul National University Bundang Hospital, Seongnam-si, Kyonggi-do, Republic of Korea; Seoul National University Children’s Hospital, Seoul, Seoul-t'ukpyolsi, Republic of Korea; Seoul National University Children’s Hospital, Seoul, Seoul-t'ukpyolsi, Republic of Korea; Seoul National University Bundang Hospital, Seongnam-si, Kyonggi-do, Republic of Korea; Seoul National University Bundang Hospital, Seongnam-si, Kyonggi-do, Republic of Korea; Seoul National University Bundang Hospital, Seongnam-si, Kyonggi-do, Republic of Korea; Seoul National University Bundang Hospital, Seongnam-si, Kyonggi-do, Republic of Korea; Seoul National University Bundang Hospital, Seongnam-si, Kyonggi-do, Republic of Korea; Seoul National University Bundang Hospital, Seongnam-si, Kyonggi-do, Republic of Korea; Seoul National University Bundang Hospital, Seongnam-si, Kyonggi-do, Republic of Korea; Seoul National University Bundang Hospital, Seongnam-si, Kyonggi-do, Republic of Korea; Seoul National University Bundang Hospital, Seongnam-si, Kyonggi-do, Republic of Korea; Seoul National University Bundang Hospital, Seongnam-si, Kyonggi-do, Republic of Korea; Seoul National University Bundang Hospital, Seongnam-si, Kyonggi-do, Republic of Korea; Seoul National University Bundang Hospital, Seongnam-si, Kyonggi-do, Republic of Korea; Seoul National University Bundang Hospital, Seongnam-si, Kyonggi-do, Republic of Korea; Seoul National University Bundang Hospital, Seongnam-si, Kyonggi-do, Republic of Korea; Seoul National University Bundang Hospital, Seongnam-si, Kyonggi-do, Republic of Korea; Seoul National University Bundang Hospital, Seongnam-si, Kyonggi-do, Republic of Korea; Seoul National University Bundang Hospital, Seongnam-si, Kyonggi-do, Republic of Korea; Seoul National University Bundang Hospital, Seongnam-si, Kyonggi-do, Republic of Korea

## Abstract

**Background:**

Carbapenems are broad spectrum antibiotics widely used for treatment of various infectious disease such as pneumonia, sepsis, and urinary tract infection. Carbapenems have been linked to a possible drug interaction which can lead to subtherapeutic levels of valproate and increase the risk of patients experiencing seizures. In this study, we reviewed cases of concomitant use of valproic acid and carbapenems, and investigated the impact of drug-drug interactions and effects.

**Methods:**

The electronic medical records of patients treated with carbapenems and valproic acid at Seoul National University Bundang Hospital from 2017 to 2022 were reviewed.

**Results:**

During the study period, a total of 183 patients were prescribed concomitantly with valproic acid and carbapenems. The average age was 64 years, 172 (94%) were >18 years of age and 11 (6%) were ≤ 18 years. The most common reason for carbapenem prescription were urinary tract infection (48.1%), pneumonia (38.3%), sepsis (24.6%), and intra-abdominal infection (5.5%). Among carbapenems, ertapenem was most common by 61.8%, followed by meropenem (39.9%), and imipenem (3.3%). Of the total cases, valproic acid monotherapy accounted for 42.6% and concomitant use with other anticonvulsants accounted for 57.4%. During concomitant use of carbapenem and valproic acid, 35.0% of patients were consulted to the infectious diseases or neurology departments about drug interactions, and 44.8% monitored the therapeutic drug concentration. During the concomitant treatment, valproic acid dosage was increased in 19.1% and other anticonvulsants were added in 12.0%. Seizure occurred in 15 cases (8.2%) of the total cases.

**Conclusion:**

As a result of investigating drug-drug interactions between carbapenem and valproic acid, serum concentrations of valproic acid were significantly decreased, some of which actually led to convulsions. Care should be taken when administering carbapenem to patients receiving valproic acid. If concomitant administration is unavoidable, it is important to monitor serum concentrations and consider alternative anticonvulsants. Continuing education and monitoring of therapeutic drug concentrations are required for the concomitant administration of carbapenem antibiotics and valproic acid.

**Disclosures:**

**All Authors**: No reported disclosures

